# Analysis of prognostic factors and nomogram construction for postoperative survival of triple-negative breast cancer

**DOI:** 10.3389/fimmu.2025.1561563

**Published:** 2025-04-07

**Authors:** Chenxi Wang, Xiangqian Zhao, Dawei Wang, Jinyun Wu, Jizhen Lin, Weiwei Huang, Yangkun Shen, Qi Chen

**Affiliations:** ^1^ Fujian Key Laboratory of Innate Immune Biology, Biomedical Research Center of South China, College of Life Science, Fujian Normal University, Fuzhou, Fujian, China; ^2^ The Cancer Center, Fujian Medical University Union Hospital, Fuzhou, Fujian, China; ^3^ Department of Medical Oncology, Clinical Oncology School of Fujian Medical University, Fujian Cancer Hospital, Fujian Provincial Key Laboratory of Translational Cancer Medicine, Fuzhou, Fujian, China

**Keywords:** triple-negative breast cancer, SEER database, prognostic modelling, columnar plots, machine learning

## Abstract

**Introduction:**

Triple-negative breast cancer (TNBC) is a highly aggressive breast cancer subtype associated with poor prognosis and limited treatment options. This study utilized the SEER database to investigate clinicopathologic characteristics and prognostic factors in TNBC patients.

**Methods:**

Machine learning algorithms specifically Gradient Boosting Machines (XGBoost) and Random Forest classifiers were applied to develop survival prediction models and identify key prognostic markers.

**Results:**

Results indicated significant predictors of survival, including tumor size, lymph node involvement, and distant metastases. Our proposed work showed better predictive performance, with a C-index of 0.8544 and AUC-ROC values of 0.9008 and 0.8344 for one year and three year overall survival predictions. Major predictors of survival comprises tumor size, HR is 3.657 for T4, lymph node involvement, HR is 3.018 for N3, distant metastases, HR is 1.743 for M1, and prior treatments includes surgery, HR is 0.298, chemotherapy, HR is 0.442, and radiotherapy, HR is 0.607.

**Discussion:**

The findings emphasize the clinical utility of AI-driven models in improving TNBC prognosis and guiding personalized treatment strategies. This study provides novel insights into the survival dynamics of TNBC patients and underscores the potential of predictive analytics in oncology.

## 1 Introduction

Breast cancer is the most prevalent form of cancer among women and the primary cause of cancer-related mortality among women in the majority of countries (1). A significant proportion of breast cancer patients, estimated to be between 15% and 20%, are diagnosed with triple-negative breast cancer (TNBC) (2). The absence of intrinsic biomarkers renders endocrine therapies and conventional HER2-targeted therapies ineffective (3). TNBC is highly heterogeneous, and the lack of standard therapeutic protocols results in the disease being prone to metastasis in the early stages, with a higher risk of recurrence and therefore a poor prognosis (4). Epidemiological data indicate that the five-year survival rate following a diagnosis of TNBC is 77%, in comparison to 91% for other forms of breast cancer (5).

Once a diagnosis of triple-negative breast cancer (TNBC) has been established, surgical excision of the tumor in conjunction with chemotherapy and/or radiotherapy represents the prevailing standard of care. Perioperative chemotherapy encompasses both neoadjuvant chemotherapy and postoperative conventional chemotherapy. TNBC has been shown to exhibit relative sensitivity to perioperative chemotherapy, with proven efficacy, thereby conferring a certain degree of group benefit to patients. However, in patients with advanced and recurrent TNBC, chemotherapy has limited effectiveness and provides only minimal benefit (6). In recent years, researchers have been working to make breakthroughs in the field of TNBC treatment. Emerging therapeutic approaches include chemotherapy combined with immunotherapy and angiogenesis inhibition therapy. Immunotherapy regulates the tumor microenvironment through the use of pharmacological agents, enhancing the anti-tumor activity of immune cells and preventing tumor progression. This approach has been shown to have precise efficacy and significant benefits. Current evidence strongly supports the use of immune checkpoint inhibitors in combination with chemotherapy for the neoadjuvant treatment of TNBC and recurrent metastases (7–9).

Additionally, research into PAM pathway-targeted drugs, androgen receptor antagonists, and angiogenesis inhibitors has shown potential for improving TNBC prognosis. Among these, angiogenesis inhibitors and antibody-drug conjugates (ADCs) have gained significant attention for their ability to enhance survival in metastatic TNBC [10-12]. Despite these advancements, most prognostic studies on TNBC remain retrospective, and the prognostic risk factors influencing overall survival in TNBC patients are not well-defined. This gap underscores the need for predictive models that integrate clinical and biological data to guide treatment decisions.

Explainable AI (XAI) and Machine Learning techniques can play a critical role in advancing TNBC prognosis and treatment planning. XAI ensures that predictive models provide transparent, interpretable insights into the key factors affecting survival, facilitating clinical decision-making. Machine Learning algorithms, when applied to large datasets like the SEER database, enable the identification of complex patterns and interactions that traditional statistical methods may overlook. These techniques offer a robust framework for developing individualized treatment plans while maintaining the interpretability required for clinical practice.

For this study, 23,729 patients with TNBC were screened using the SEER database for the years 2018 to 2021. This timeframe aligns with current treatment paradigms, enabling the generation of clinically relevant insights. Systemic therapy has been established as the standard treatment option for patients with advanced breast cancer at initial diagnosis. Among patients with advanced triple-negative breast cancer (TNBC), systemic chemotherapy is a common treatment. Although local management of the primary lesion, such as palliative resection, is often employed, there is still considerable debate within the academic community as to whether local treatment can significantly improve long-term survival in patients with stage IV TNBC (10). Furthermore, patients with advanced TNBC frequently present with symptoms such as bone metastases and local compression, necessitating the use of radiotherapy as a crucial treatment option in such cases (11). To investigate the prognostic impact of different treatment strategies, this study conducted a comparative analysis of survival data from patients recorded in the SEER database and those admitted to Fujian Cancer Hospital. The study also integrated data on metastatic sites and treatments received, including surgery, radiotherapy, and chemotherapy, to evaluate their impact on patient outcomes. Column charts, as graphical assessment tools, quantify risk based on statistical prediction models. Evidence suggests that the column chart not only serves as an alternative to the AJCC TNM staging system but also surpasses the traditional TNM staging system in terms of individualized predictive ability making them reliable tools for oncology prognosis (12–14). This study constructed and validated a column-line graph using the SEER database to predict survival probabilities for TNBC patients. The survival prediction model was further enhanced by incorporating Explainable AI and Machine Learning techniques, followed by analyses to evaluate performance, validate predictive accuracy, and determine the significance of key features.

This study, utilizing Explainable AI (XAI) and Machine Learning (ML), can enhance personalized treatment planning, early diagnosis, and precision oncology by identifying high-risk patients and optimizing therapeutic strategies. It also supports systemic therapy optimization, advances tumor microenvironment research, and extends predictive modeling to other cancers. This work makes significant advancements in predictive models for TNBC by employing sophisticated techniques in machine learning and explainable artificial intelligence (XAI). These methods enhance both the accuracy of predictions and the interpretability of results, offering a marked improvement over conventional statistical models. We utilized SHAP values to effectively identify crucial prognostic factors that can influence treatment outcomes, thereby facilitating more accurate assessments of patient risk. Additionally, it informs health policy for better resource allocation and integrates with clinical decision support systems, empowering clinicians to improve long-term patient outcomes and survivorship care.

The proposed work advances beyond existing works by leveraging advanced ML algorithm XGBoost and Random Forest integrated with Explainable AI (XAI) methods (SHAP and LIME) to enable interpretable predictions, enhancing clinical decision-making. With the help of 23,729 TNBC patient records extracted from the SEER database and an extension cohort, our work captures present treatment paradigms, confirming clinically proved insights. This survival prediction models shows better accuracy, with a C-index of 0.8544 and AUC-ROC values of 0.9008 and 0.8344 for one year and three year survival predictions, surpassing conventional prognostic techniques. Through SHAP analysis, we predicted the major influential predictors of survival including tumor size, lymph node involvement, metastases, and treatments, delivering actionable insights for personalized treatment planning. By addressing these existing research gaps, our work offers a strong and interpretable framework for enhancing TNBC prognosis, guiding treatment decisions, and upgrading precision oncology. However, relying solely on SEER data may limit the applicability of findings to diverse populations. To address this limitation, we also added an extension cohort of 163 stage IV TNBC patients from Fujian Cancer Hospital, providing insights into clinical outcomes within an Asian population. This dual-dataset method enables the validation of suggested ML algorithms over various demographic and healthcare contexts, strengthening the robustness and cross-population applicability of our proposed prognostic framework. In addition to this, we compare different survival patterns and treatment responses between these cohorts offers a broader idea on TNBC prognosis, supporting more detailed and personalized treatment techniques.

We also leverage Explainable AI (XAI) and ML algorithms to address the conventional statistical models limitations in predicting TNBC prognosis. Conventional approaches such as Cox regression approaches and Kaplan-Meier survival analysis are good and efficient for predicting survival probabilities but they depend on linear assumptions and may not be good fit for nonlinear complex relationships among clinical variables, but our XGBoost and Random Forest are good at capture high-dimensional interactions and nonlinear patterns without predefined initial assumptions, improving predictive accuracy. Moreover, XAI tools such as SHapley Additive exPlanations and Local Interpretable Model-agnostic Explanations provide interpretability by highlighting the individual contribution of each feature like tumor size, lymph node involvement, and distant metastases—to survival predictions. This interpretability is vital in clinical settings, where understanding the specific reason behind predictions is critical for gaining clinicians’ trustworthiness. By combing the above said approaches, our work is not only enhances the accuracy of TNBC survival predictions but also offers transparent insights into key prognostic factors, assisting personalized treatment decisions and advancing recent precision oncology.

## 2 Materials and methods

### 2.1 Study population

**Figure 1:**
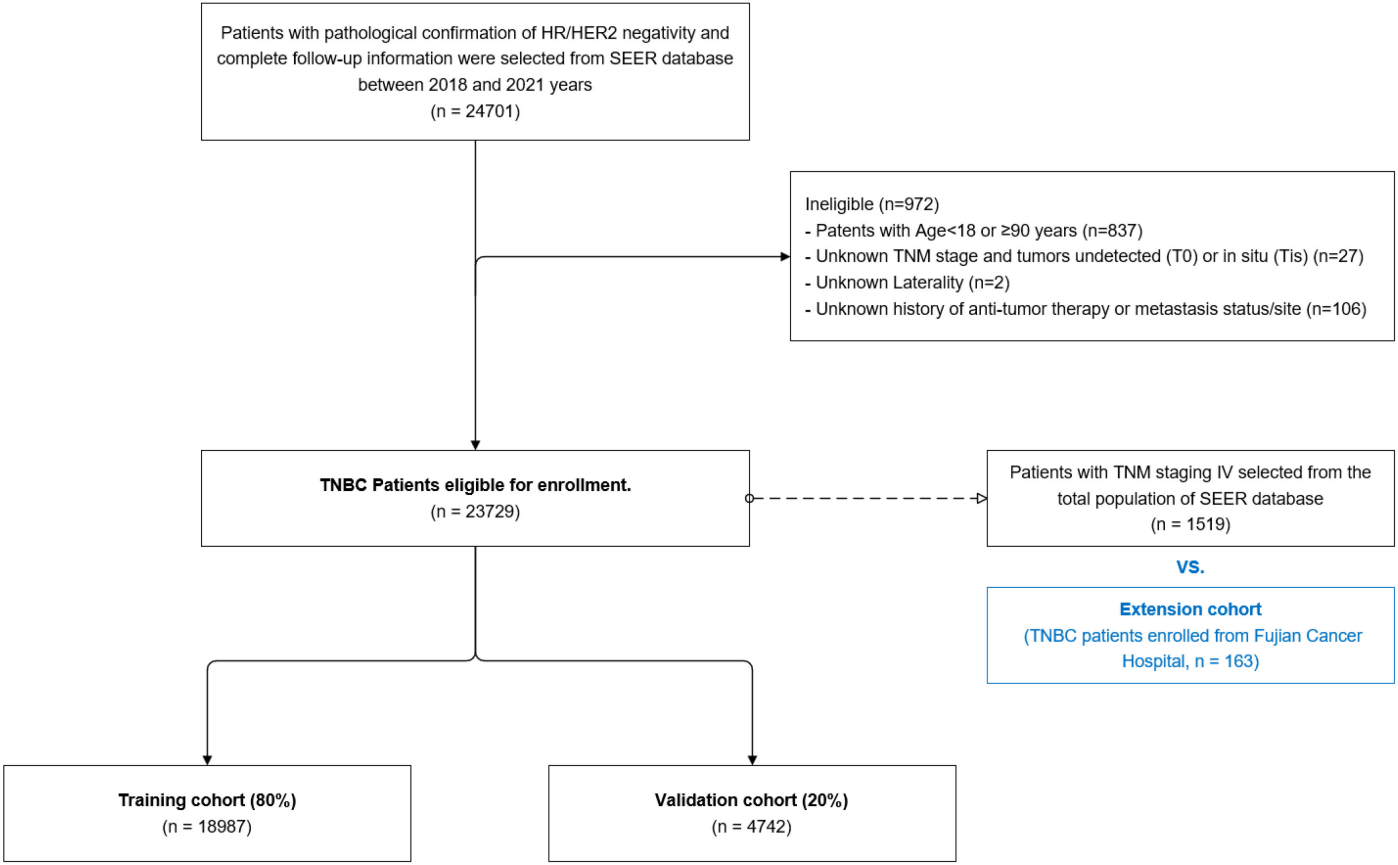
Workflow diagram illustrating data extraction, preprocessing, and model development.

A total of 23,729 patients with triple-negative breast cancer (TNBC) diagnosed from 2018 to 2021 were identified in the SEER database, and the relevant clinicopathologic data were extracted. In order to be eligible for inclusion in the study, patients were required to have undergone pathological confirmation of ER, PR, and HER2 negativity, as well as to have complete follow-up information. HER2/neu-negative was defined as either immunohistochemistry (IHC) 0–1+or IHC 2+fluorescence *in situ* hybridization (FISH)-negative, while ER/PR-negative was defined as ER/PR staining less than 1%. The exclusion criteria were as follows: age <18 years or >90 years, tumors undetected (T0) or *in situ* (Tis), absence of ultrasonography findings, and unknown laterality, TNM stage, history of anti-tumor therapy, or metastasis status/site. Furthermore, an extension cohort was established, comprising 163 patients with late-stage (IV) TNBC who were enrolled from the Medical Oncology Department of the Clinical Oncology School of Fujian Medical University, Fujian Cancer Hospital, between October 2011 and January 2023. The aforementioned patients were subject to the same inclusion and exclusion criteria. This study was approved by the Ethics Committee of Fujian Cancer Hospital and was conducted in accordance with the principles set forth in the Helsinki Declaration. All participants provided informed consent in accordance with the ethical standards set forth in the informed consent form (ICF). To ensure the reliability of our findings, we conducted an internal validation using a split of the dataset, which allowed us to rigorously assess the performance of the model. We analyzed the model’s accuracy through calibration curves and calculated area under the receiver operating characteristic (AUC-ROC) metrics. **Figure 1** illustrates the study design workflow.

### 2.2 Data collection

Information was gathered on the following clinicopathologic variables: age, race, laterality, TNM stage, surgical intervention on the primary tumor, radiation therapy on the primary tumor, chemotherapy, and distant metastases.

### 2.3 Machine learning-based prognostic modeling

Advanced machine learning (ML) techniques were employed to predict survival outcomes and identify key prognostic factors for triple-negative breast cancer (TNBC). A supervised learning approach was adopted, leveraging both clinical and demographic data extracted from the SEER database and the Fujian Cancer Hospital cohort. The ML pipeline included data preprocessing, feature selection, model training, and validation. Missing values were handled using multiple imputation techniques to minimize bias and improve data reliability. Continuous variables, such as age and TNM stage, were standardized, while categorical variables, like treatment modalities, were one-hot encoded. Patients were split into training (70%) and validation (30%) sets, ensuring balanced representation of stages and treatments.

#### 2.3.1 Feature selection using explainable AI

Feature importance was determined using SHAP (SHapley Additive exPlanations) values, providing insights into the contribution of variables such as age, TNM stage, metastasis site, and treatment modalities to survival predictions. SHAP-based analysis helped prioritize features, enhancing the model’s interpretability. The reason for selecting SHAP is its capacity to provide clear, interpretable explanations for the predictions generated by our machine learning model. This feature is essential for ensuring transparency in the identification of important prognostic factors, which can guide clinical decision-making. To improve the interpretability of the suggested ML algorithms for clinical usage, we utilized SHAP and LIME. SHAP quantifies each feature’s contribution to predictions, activating both global and patient-specific insights. For example, it highlighted surgical resection, chemotherapy, and lymphatic metastasis as key survival metrics, with visual plots providing transparent feature significance. LIME complements this by addressing individual predictions via local perturbations, pointing critical variables like TNM stage or distant metastases that classify a patient as high-risk. Together, SHAP and LIME enhance predictive accuracy while guaranteeing interpretable, actionable insights for personalized clinical decision-making.

#### 2.3.2 Model training and evaluation

Gradient Boosting Machines (e.g., XGBoost) and Random Forest classifiers were trained to predict survival outcomes (short-term vs. long-term survival). XGBoost was chosen over alternative algorithms because of its demonstrated efficiency in managing large, imbalanced datasets and its ability to effectively capture complex nonlinear relationships within the data. Hyperparameter tuning was performed using Bayesian optimization to achieve optimal model performance. Model evaluation metrics included accuracy, precision, recall, F1-score, and area under the receiver operating characteristic curve (AUC-ROC).

#### 2.3.3 Validation and explainability

The performance of the predictive models was validated using the test dataset, with results visualized through calibration curves and confusion matrices. Local Interpretable Model-agnostic Explanations (LIME) were employed alongside SHAP to validate individual predictions, enabling a granular understanding of the impact of specific patient attributes on survival outcomes.

#### 2.3.4 Kaplan-Meier survival analysis with AI integration

Kaplan-Meier survival curves were enriched with ML predictions to evaluate the consistency between AI-derived risk stratification and actual survival data. Patients were grouped into high-risk and low-risk categories based on ML predictions, and survival differences were analyzed using the log-rank test.

#### 2.3.5 Ethical and transparent use of AI

To maintain ethical standards, the AI models were tested for potential biases related to demographics (e.g., ethnicity and age). The explainable models ensured transparency, fostering trust in AI-driven prognostic insights. To reduce demographic biases, we examined subgroup representation in dual datasets. The SEER cohort included 21.0% Black, 69.3% Caucasian, 9.0% Pacific Islander, and 0.7% American/Alaska/Indian Native patients. To enhance generalizability, we comprised the Fujian Cancer Hospital cohort, addressing an Asian population. Model accomplishment was examined on various factors such as age, race, and treatment groups with accuracy, AUC-ROC, and calibration curves, with SHAP values guaranteeing consistent feature importance. Stratified sampling of 80% training and 20% validation-maintained subgroup proportions, fend off overfitting and guarantee reliable predictions over the diversified profiles.

### 2.4 Statistical analysis

In this study, 23,729 patients were randomly divided into a training cohort (n = 18,987, 80%) and a validation cohort (n = 4,742, 20%). The training cohort was used to construct a prognostic model, while the validation cohort provided external validation. Cox regression analyses identified factors associated with overall survival (OS), and hazard ratios (HRs) with 95% confidence intervals (CIs) were calculated. A prognostic nomogram for 1-year and 3-year survival probabilities was developed, with model performance assessed using the C-index and calibration curves. Survival time was estimated using the Kaplan–Meier method and log-rank tests. Statistical analyses were performed using R v4.3.2, with p < 0.05 considered significant. A p-value threshold of <0.05 was opted to measure statistical importance, related to the standard practices in medical investigations. This threshold value balances the risk of Type I errors while maintaining considerable sensitivity to identify meaningful associativity’s. Given the multiple comparisons in our detailed investigation, the threshold was pertained constantly to preserve interpretability without overly inflating the risk of false positives. This way guarantees that highlighted prognostic factors and model performance metrics are both statistically strong and clinically related.

Our rigorous multivariate analysis has confirmed the independent protective effect of surgical intervention, as evidenced by a statistically lower hazard ratio for mortality. These findings are in accordance with established literature in the field, further supporting the clinical benefits of surgical treatments for TNBC and aiding healthcare professionals in making informed decisions regarding patient candidate selection for operative management.

## 3 Results

### 3.1 Population characteristic

This study identified 23,729 triple-negative breast cancer (TNBC) patients from the SEER database. The cohort had a median age of 59.89 years, predominantly comprising Caucasian patients (69.0%). Detailed baseline characteristics, including TNM staging, treatment modalities, and demographic information, are presented in **Table 1**. Histological grading was excluded from analysis due to missing data.

**Table 1:** Patient baseline demographic and clinical characteristics.

	Training cohort (n=18987)	Validation cohort (n=4742)	Total patients (n=23729)	*P*
Age	59.93 (14.0)	59.73 (13.6)	59.89 (13.92)	*0.373*
Race				
White	13177 (69.4)	3269 (68.9)	16446 (69.3)	*0.933*
Black	3982 (21.0)	1005 (21.2)	4987 (21.0)	
Asian or Pacific Islander	1700 (9.0)	435 (9.2)	2135 (9.0)	
American Indian/Alaska Native	128 (0.7)	33 (0.7)	161 (0.7)	
Laterality				
Left	9767 (51.4)	2460 (51.9)	12227 (51.5)	*0.756*
Right	9213 (48.5)	2281 (48.1)	11494 (48.4)	
Bilateral	7 (0.0)	1 (0.0)	8 (0.0)	
T stage				
T1	7951 (41.9)	2048 (43.2)	9999 (42.1)	*0.182*
T2	8111 (42.7)	2004 (42.3)	10115 (42.6)	
T3	1671 (8.8)	411 (8.7)	2082 (8.8)	
T4	1254 (6.6)	279 (5.9)	1533 (6.5)	
N stage				
N0	12672 (66.7)	3195 (67.4)	15867 (66.9)	*0.179*
N1	4738 (25.0)	1197 (25.2)	5935 (25.0)	
N2	721 (3.8)	168 (3.5)	889 (3.7)	
N3	856 (4.5)	182 (3.8)	1038 (4.4)	
M stage				
M0	17781 (93.6)	4429 (93.4)	22210 (93.6)	*0.553*
M1	1206 (6.4)	313 (6.6)	1519 (6.4)	
TNM stage				
I	6695 (35.3)	1730 (36.5)	8425 (35.5)	*0.280*
II	6315 (33.3)	1557 (32.8)	7872 (33.2)	
III	4771 (25.1)	1142 (24.1)	5913 (24.9)	
IV	1206 (6.4)	313 (6.6)	1519 (6.4)	
Surgery on primary tumor				
Yes	17055 (89.8)	4256 (89.8)	21311 (89.8)	*0.902*
No	1932 (10.2)	486 (10.2)	2418 (10.2)	
Radiation				
Yes	9888 (52.1)	2476 (52.2)	12364 (52.1)	*0.879*
No	9099 (47.9)	2266 (47.8)	11365 (47.9)	
Chemotherapy				
Yes	15013 (79.1)	3795 (80.0)	18808 (79.3)	*0.150*
No	3974 (20.9)	947 (20.0)	4921 (20.7)	
Location of metastases				
Bone	516 (2.7)	138 (2.9)	654 (2.8)	*0.500*
Brain	141 (0.7)	42 (0.9)	183 (0.8)	*0.360*
Liver	321 (1.7)	76 (1.6)	397 (1.7)	*0.720*
Lung	481 (2.5)	116 (2.4)	597 (2.5)	*0.771*
Lymph node	496 (2.6)	122 (2.6)	618 (2.6)	*0.919*
Other	229 (1.2)	54 (1.1)	283 (1.2)	*0.759*

The majority of patients were categorized as T1 (42.1%) and T2 (42.6%), with N0 (66.9%) as the predominant nodal classification. A balanced distribution was observed in AJCC staging, with Stage I (35.5%) and Stage II (33.2%) patients. Treatment patterns showed that 89.8% underwent surgery, while 79.3% received chemotherapy, and 52.1% underwent radiotherapy. These findings indicate aggressive management strategies for early-stage TNBC, emphasizing systemic and localized interventions (see **Table 1** for comprehensive data). For patients with stage IV TNBC (n = 1,519), the median age was slightly higher at 60.56 years. A smaller fraction (27.3%) underwent surgical treatment, consistent with palliative care principles in advanced disease. However, 80.2% received chemotherapy, and 28.9% underwent radiotherapy. The clinicopathological characteristics of this subgroup are detailed in **Table 2**, highlighting a shift toward systemic treatments in metastatic TNBC.

**Table 2:** Demographics and clinical characteristics of the patients in extension cohort (comparing with subgroup of TNM staging IV in SEER database).

	Extension cohort (n=163)	TNM-IV (SEER) (n=1519)	*P*
Age	49.67 (9.6)	60.56 (14.32)	*<0.001*
Surgery on primary tumor			
Yes	120 (73.6)	413 (27.3)	*<0.001*
No	43 (26.4)	1106 (72.8)	
Radiation			
Yes	42 (25.8)	439 (28.9)	*0.400*
No	121 (74.2)	1080 (71.1)	
Chemotherapy			
Yes	160 (98.2)	1218 (80.2)	*<0.001*
No	3 (1.8)	301 (19.8)	
Location of metastases			
Bone	68 (41.7)	654 (43.1)	*0.743*
Brain	68 (5.5)	183 (12.0)	*<0.001*
Liver	31 (19.0)	397 (26.1)	*0.0474*
Lung	67 (41.1)	597 (39.3)	*0.655*
Lymph node	116 (71.2)	618 (40.7)	*<0.001*
Other	24 (14.7)	283 (12.0)	*0.220*

In contrast, the extension cohort of 163 metastatic TNBC patients from Fujian Cancer Hospital exhibited a younger median age (49.67 years). A higher proportion underwent surgery (73.6%) and chemotherapy (98.2%), with fewer receiving radiotherapy (25.8%). These differences may reflect regional variations in healthcare practices, patient selection criteria, or treatment approaches (see **Table 2**).

**Figure 2:**
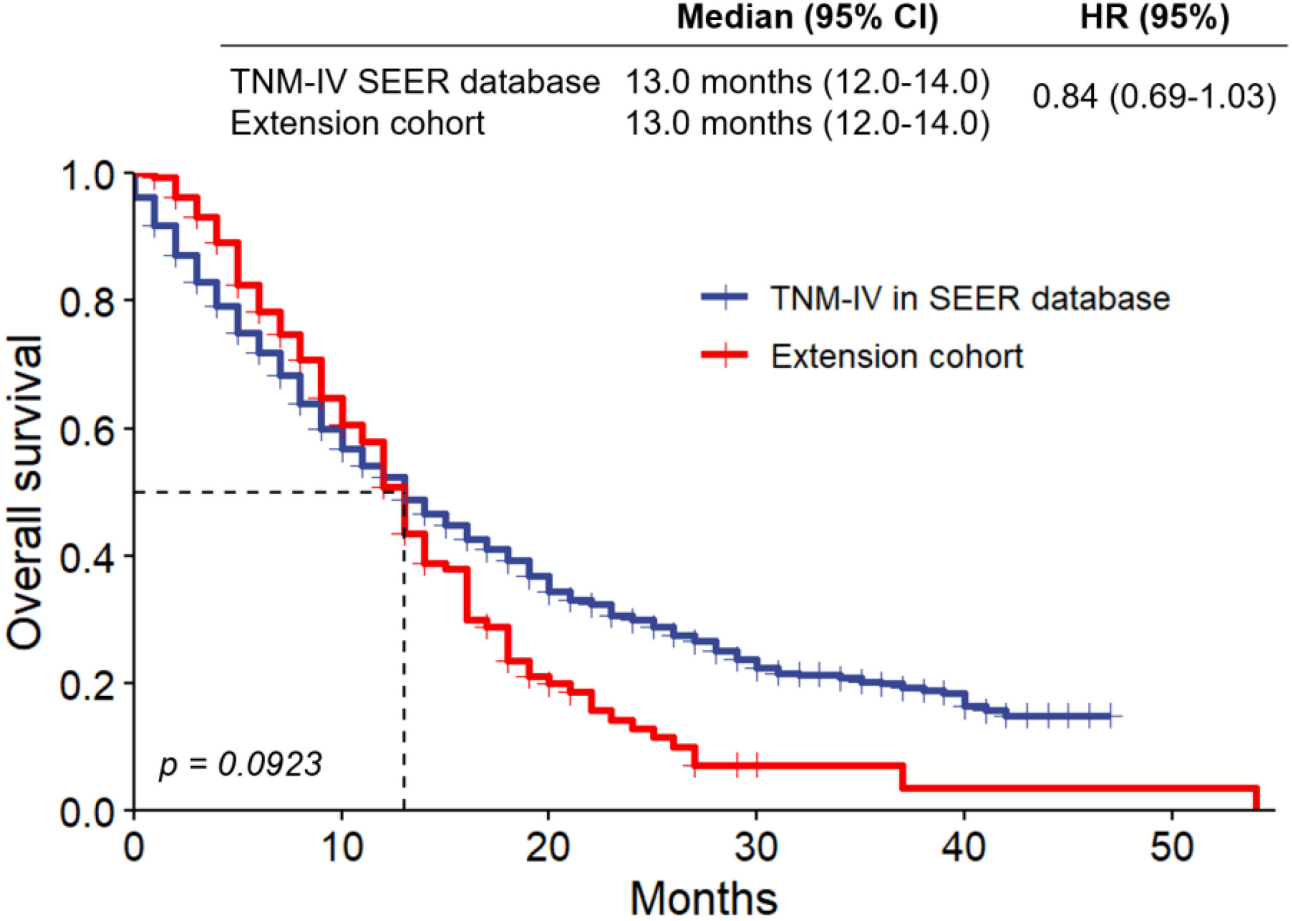
Kaplan-Meier survival curves stratified by tumor size (≤2 cm vs. >2 cm). Analysis of overall survival in extension cohort and the subgroup of patients with TNM staging IV selected from SEER database. HR, hazard ratio.

Survival analysis using Kaplan-Meier curves (**Figure 2**) revealed no significant difference in overall survival (OS) between SEER stage IV patients and the Fujian cohort (13.0 months for both groups; HR = 0.84, p = 0.0923). Despite differences in demographics and treatment strategies, these findings highlight the uniformly poor prognosis of advanced TNBC.

Our study’s clinical and demographic profiles provide significant information about how to treat TNBC. 69.3% of patients in the SEER cohort were Caucasian, 21.0% were Black, 9.0% were Asian/Pacific Islander, and 0.7% were American, Indian/Alaska Native. The median age of the group was 59.89 years. Poorer survival rates were associated to older age (≥77 years, HR: 2.42), and the importance of early identification is highlighted by the predominance of early-stage tumors (T1: 42.1%, T2: 42.6%).

**Figure 3:**
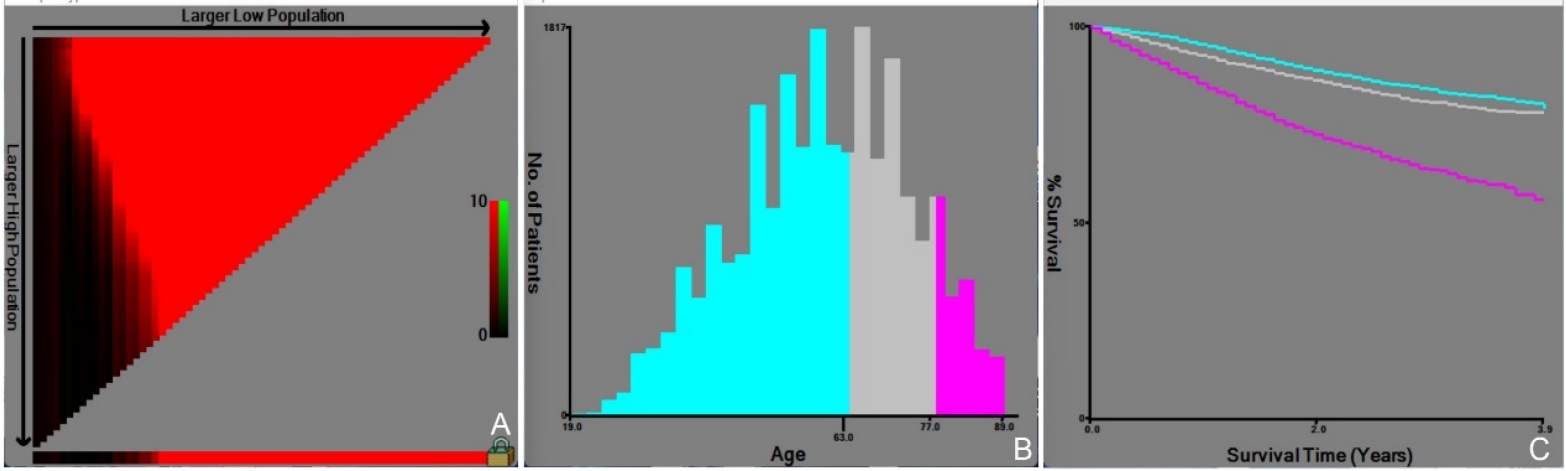
Determination of the optimal age cutoff by X-tile analysis. Appearance of the optimal grouping value for age **(A)**, Histograms of different age groups **(B)**, and Survival curves for different age groups **(C)**.

Radiotherapy (52.1%, HR: 0.607), chemotherapy (79.3%, HR: 0.442), and surgery (89.8%, HR: 0.298) were found to improve survival outcomes among the available treatment options. The Fujian cohort, on the other hand, showed greater rates of chemotherapy (98.2%) and surgery (73.6%), as well as a lower median age of 49.67 years, indicating regional variations in treatment philosophies. The optimal cut-off values for age were determined by X-Tile analysis to be 63 and 77 years. The cohort was divided into three age groups (≤63 years, 63-77 years, and ≥77 years) based on the identified cut-off values (**Figure 3**). The relative risk of the three age groups was found to be 1.00 for the ≤63-year age group, 1.27 for the 63-77-year age group, and 2.42 for the ≥77-year age group.

The impact of regional healthcare practices on TNBC outcomes is addressed by the differences between the cohorts from SEER and Fujian Cancer Hospital. China’s emphasis on surgical resection, even in advanced cases, is reflected in the Fujian cohort’s greater surgery rates (73.6% vs. 27.3% in SEER stage IV) and younger median age (49.67 vs. 59.89 years). This strategy is in accordance with regional recommendations that emphasize tumor debulking as a means of enhancing survival and reducing symptoms. The SEER cohort, on the other hand, followed typical U.S. procedures, which emphasize systemic therapy over surgery, which is less likely for stage IV TNBC. Notwithstanding these variations, stage IV patients in both cohorts had comparable overall survival rates (13.0 months, HR = 0.84, p = 0.0923), indicating that invasive surgical procedures in advanced disease may not have a significant impact on survival. These conclusions underscore the significance of tailoring treatment strategies to regional contexts while aligning with evidence-based guidelines to optimize results over the diverse populations.

### 3.2 Prognostic analysis

**Figure 4:**
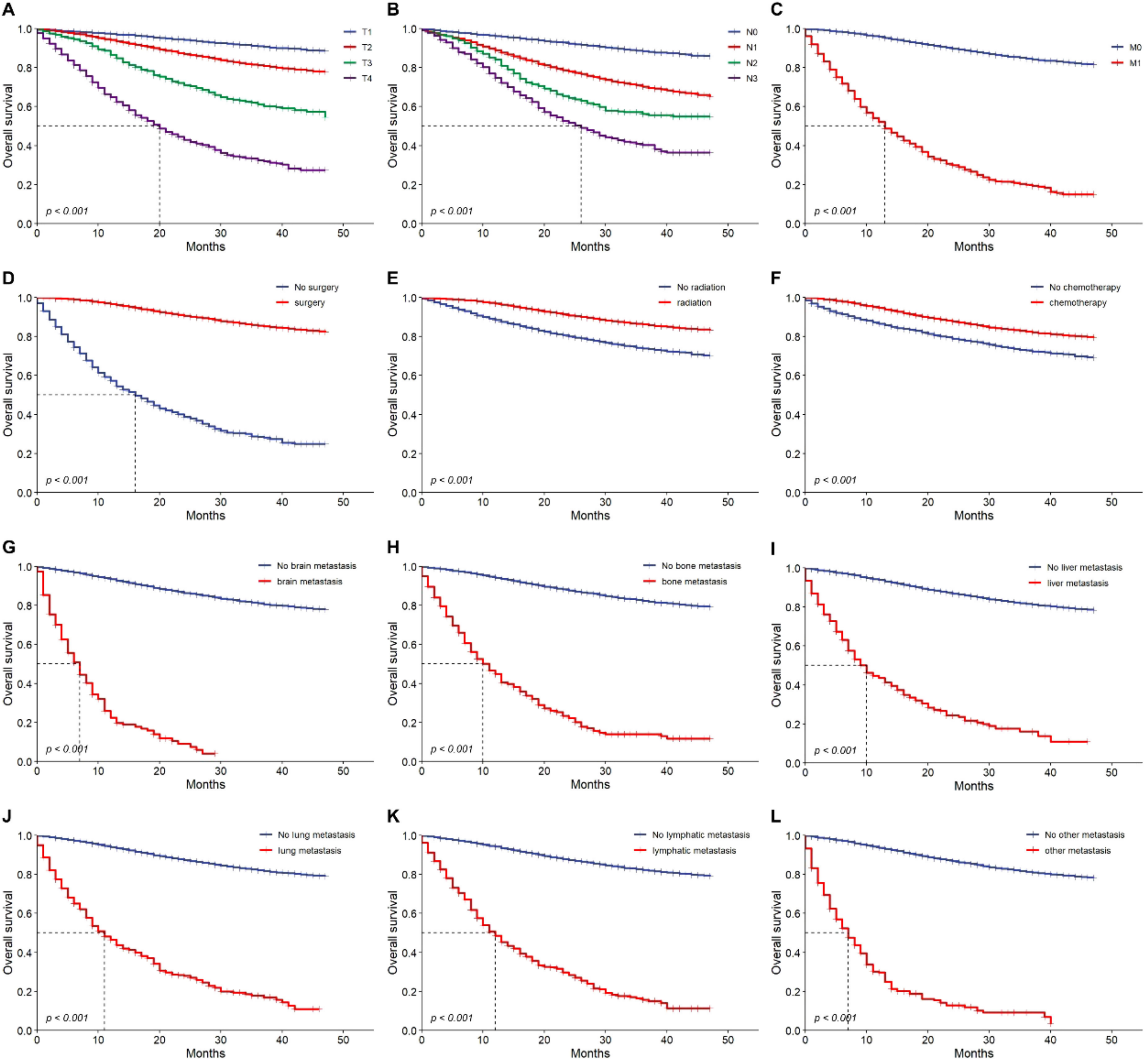
Overall survival outcomes. Kaplan-Meier analysis of survival showing overall survival in training cohort. T1~4 stage **(A)**, N0~3 stage **(B)**, M0 vs. M1 **(C)**, No previous surgery vs. previous surgery **(D)**, no radiation vs. radiation **(E)**, no chemotherapy vs. chemotherapy **(F)**, brain metastasis **(G)**, bone metastasis **(H)**, liver metastasis **(I)**, lung metastasis **(J)**, lymphatic metastasis **(K)** and other metastasis **(L)** or not. HR, hazard ratio.

Cox regression analysis identified key prognostic factors for TNBC, including advanced age (HR: 1.030, 95% CI: 1.020-1.030, p<0.001), bilateral breast cancer (HR: 7.500, 95% CI: 3.120-18.100, p<0.001), distant metastases, and advanced TNM stage, which were linked to earlier death. Longer survival was associated with previous surgery (HR: 0.089, 95% CI: 0.082-0.097, p<0.001), radiation therapy (HR: 0.423, 95% CI: 0.388-0.460, p<0.001), and chemotherapy (HR: 0.523, 95% CI: 0.480-0.570, p<0.001) (**Table 3**). Multivariate analysis confirmed that age, TNM stage, previous surgery, radiation, chemotherapy, and distant metastases (bone, brain, liver) were independently associated with prognosis (**Table 3**). Prognostic impact was further analyzed using Kaplan-Meier survival curves (**Figure 4**). We looked at the differences in surgical outcomes between TNM stages and the impact of chemotherapy on survival in patients with distant metastases in order to assess the interaction effects between important variables. In early-stage triple-negative breast cancer (TNBC), surgical resection provided the highest survival benefit; the hazard ratios (HR) for stage I, stage II, and stage III were 0.298, 0.442, and 0.607, respectively. The limited advantages of surgical intervention in advanced disease, however, are reflected in the fact that the impact of surgery decreased in stage IV patients. Similar to this, chemotherapy was crucial in treating metastatic TNBC since it dramatically increased survival in patients with bone metastases (HR: 1.173), liver metastases (HR: 1.498), and brain metastases (HR: 2.610). The significance of early intervention was further highlighted by SHAP analysis, which showed that the survival advantages of chemotherapy and surgery were more noticeable in patients with fewer metastatic locations and a lower tumor burden. In order to optimize therapeutic results, these interactions emphasize the need to customize treatment plans according to disease stage and metastatic patterns.

**Table 3:** Univariate and multivariate cox analysis of overall survival of the training cohort.

	Univariate analysis			Multivariate analysis		
	HR	95%Cl	*P*	HR	95%Cl	*P*
Age	1.030	1.020-1.030	***<0.001***	1.021	1.017-1.024	***<0.001***
Laterality						
Left	1.000			1.000		
Right	0.958	0.884-1.040	*0.302*	1.004	0.926-1.089	*0.972*
Bilateral	7.500	3.120-18.100	***<0.001***	1.167	0.478-2.846	*0.745*
T stage						
T1	1.000			1.000		
T2	2.140	1.910-2.390	***<0.001***	1.912	1.702-2.147	***<0.001***
T3	5.100	4.460-5.840	***<0.001***	3.201	2.769-3.700	***<0.001***
T4	13.000	11.500-14.700	***<0.001***	3.657	3.143-4.256	***<0.001***
N stage						
N0	1.000			1.000		
N1	2.870	2.610-3.150	***<0.001***	1.961	1.767-2.177	***<0.001***
N2	4.830	4.160-5.610	***<0.001***	3.004	2.556-3.532	***<0.001***
N3	7.400	6.540-8.370	***<0.001***	3.018	2.604-3.498	***<0.001***
M stage						
M0	1.000			1.000		
M1	12.200	11.200-13.400	***<0.001***	1.743	1.457-2.086	***<0.001***
Surgery on primary tumor						
No	1.000			1.000		
Yes	0.089	0.082-0.097	***<0.001***	0.298	0.265-0.335	***<0.001***
Radiation						
No	1.000			1.000		
Yes	0.423	0.388-0.460	***<0.001***	0.607	0.553-0.666	***<0.001***
Chemotherapy						
No	1.000			1.000		
Yes	0.523	0.480-0.570	***<0.001***	0.442	0.400-0.488	***<0.001***
Location of metastases						
Bone	12.700	11.300-14.300	***<0.001***	1.173	1.008-1.366	***0.040***
Brain	21.200	17.400-25.900	***<0.001***	2.610	2.092-3.256	***<0.001***
Liver	12.000	10.400-13.900	***<0.001***	1.498	1.268-1.771	***<0.001***
Lung	11.700	10.400-13.200	***<0.001***	1.084	0.929-1.266	*0.306*
Lymph node	10.700	9.500-12.100	***<0.001***	0.896	0.769-1.045	*0.163*
Other	17.600	15.000-20.600	***<0.001***	1.456	1.219-1.739	***<0.001***

Bold values indicate statistically significant differences.

### 3.3 Construction and calibration of nomogram to predict the prognosis of patients with breast cancer

**Figure 5:**
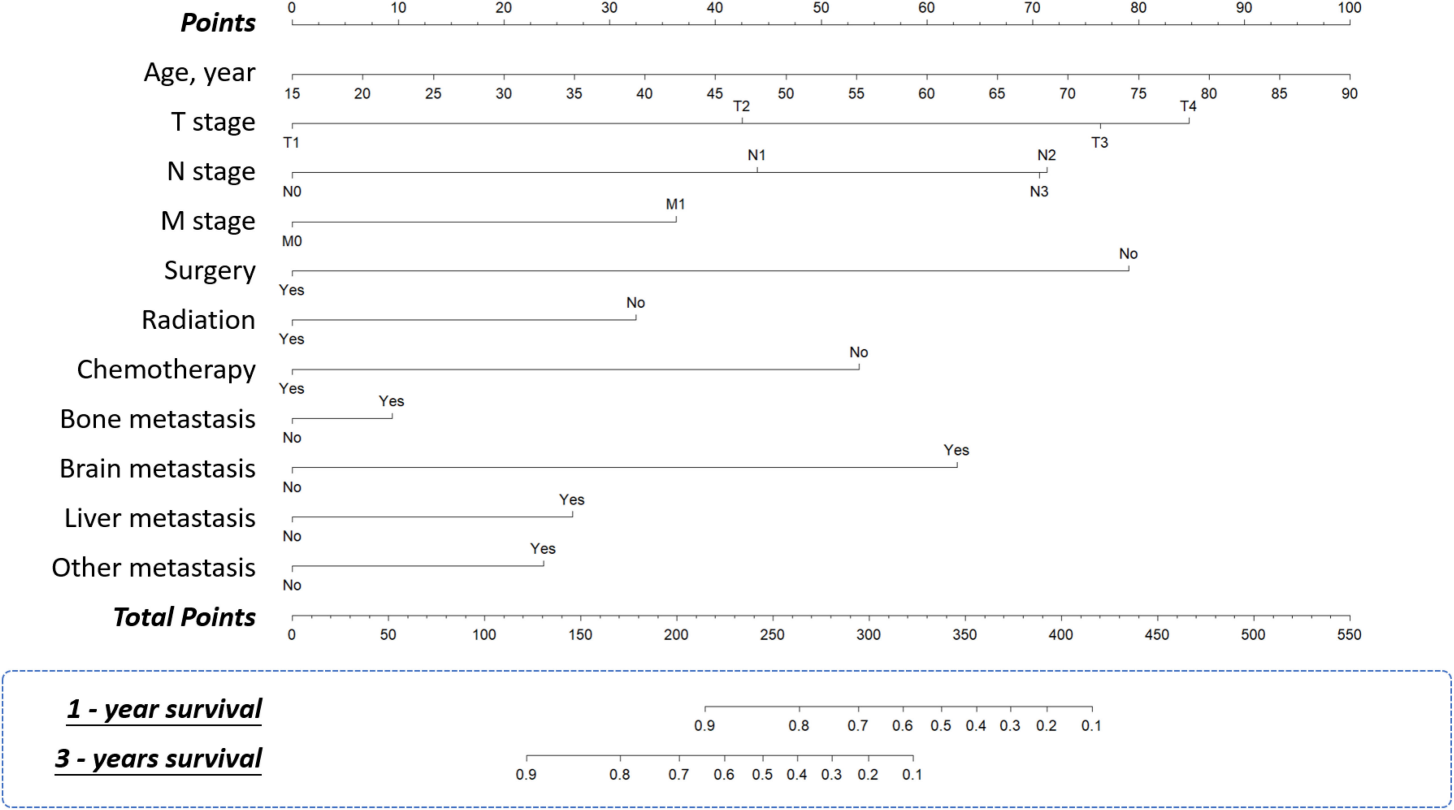
Nomograms. Nomograms for predicting 1-year and 3-year overall survival of the patients with TNBC.

**Figure 6:**
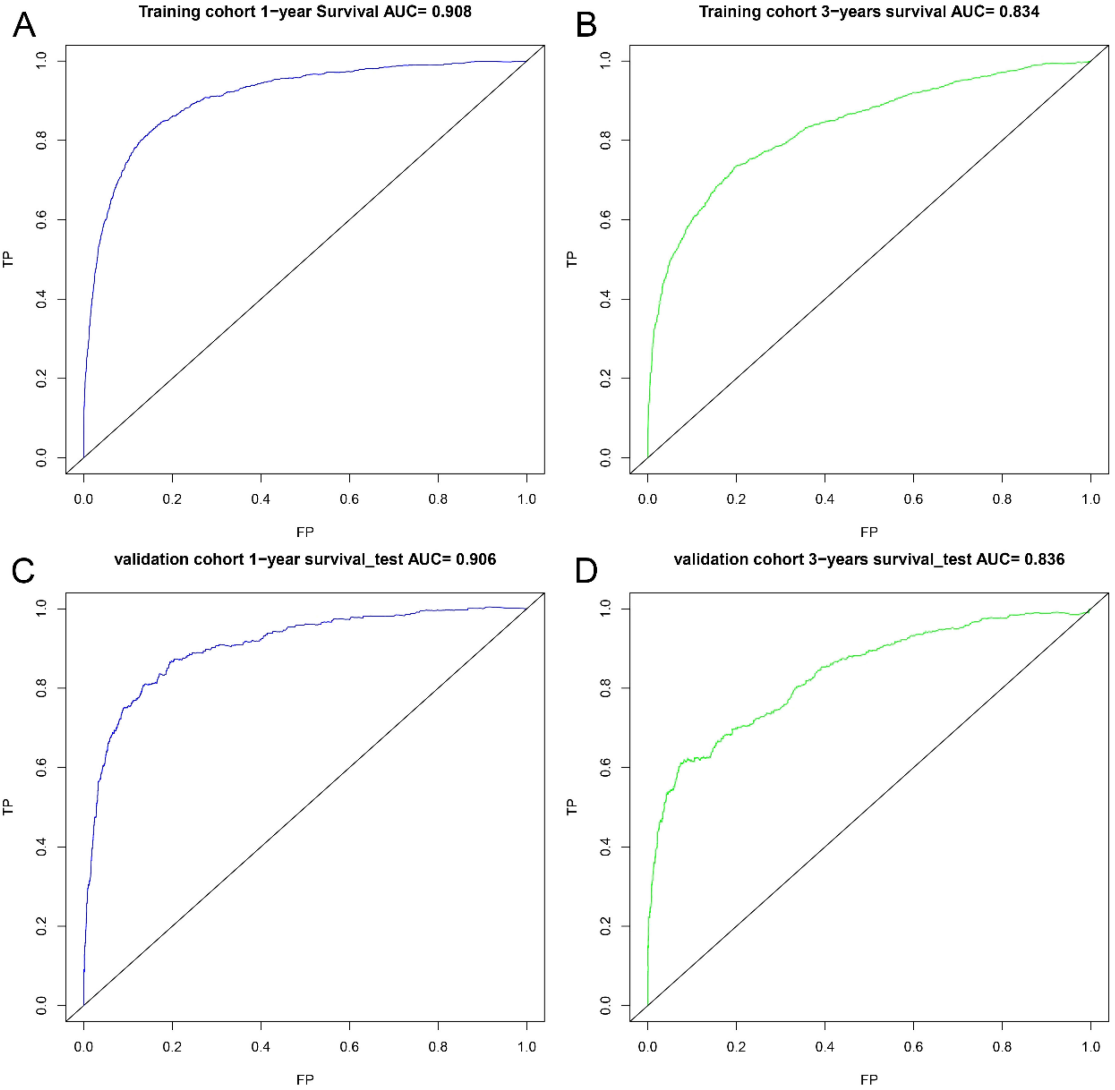
ROC curves. ROC curves for the 1-year prognostic model **(A)** and the 3-years prognostic model **(B)** in training cohort, and for the 1-years prognostic model **(C)** and the 3-years prognostic model **(D)** in validation cohort.

**Figure 7:**
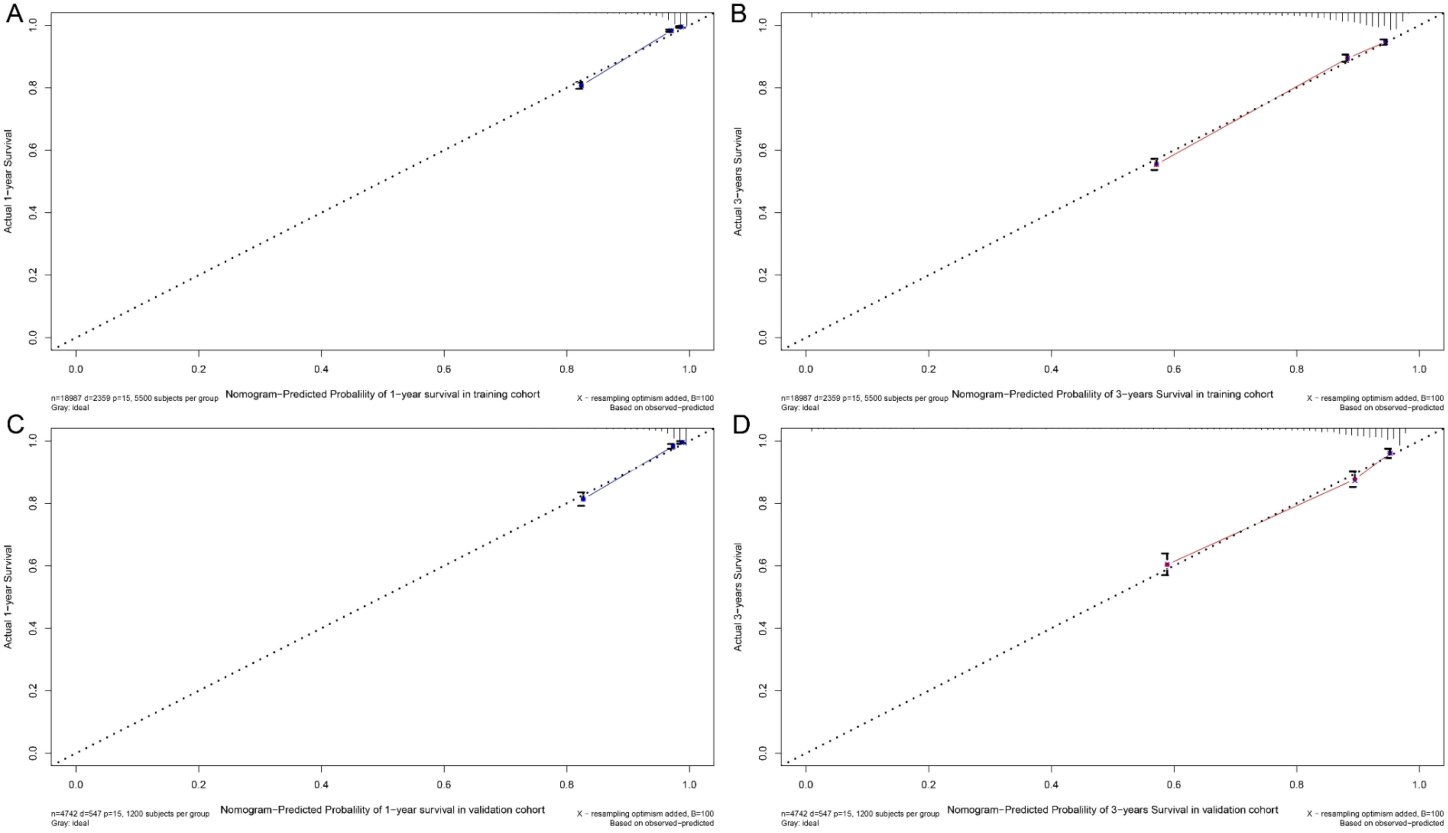
Calibration curve. Predicting patient survival at one year **(A)** and 3 years **(B)** in training cohort, and at one years **(C)** and 5 years **(D)** in validation cohort.

**Figure 8:**
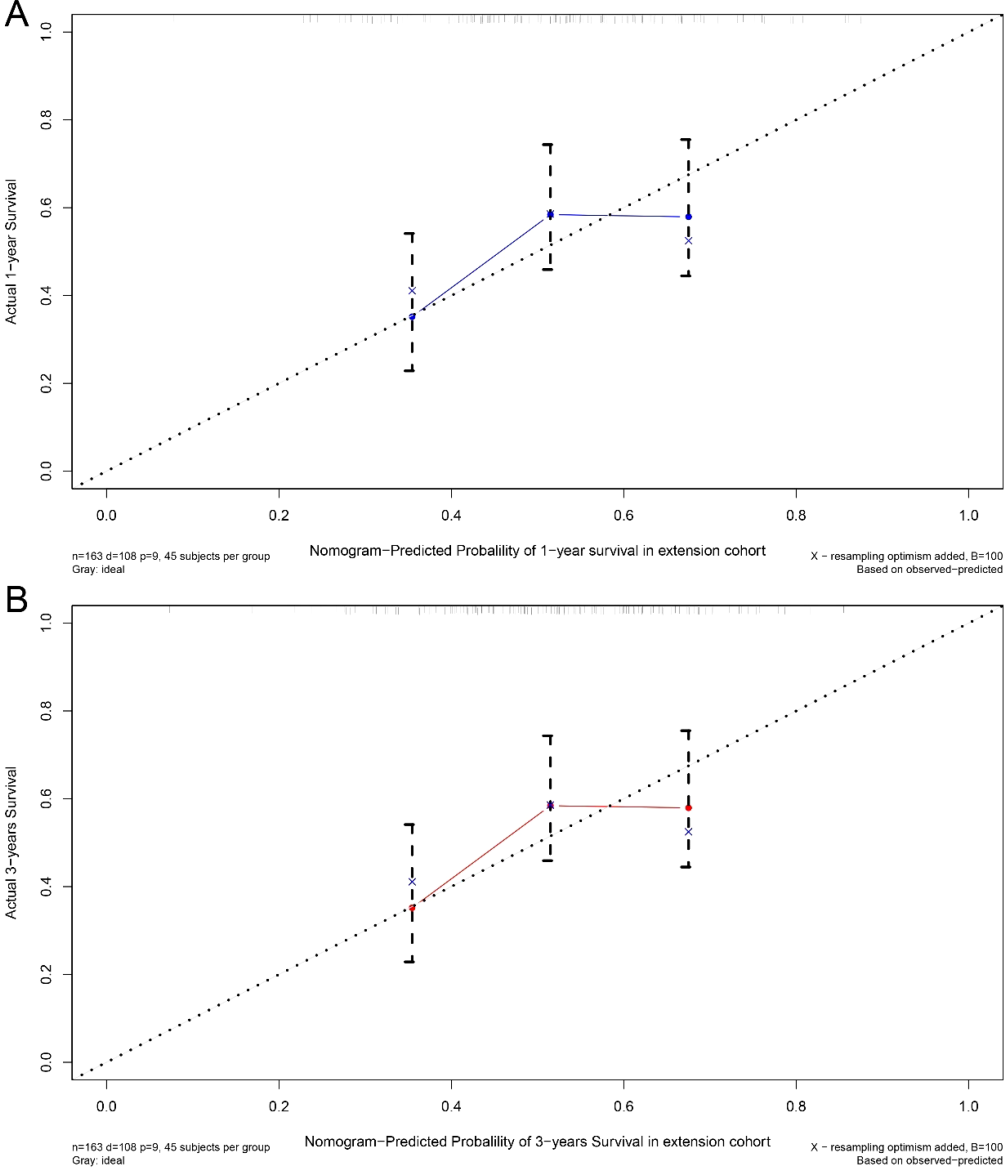
Calibration curve. Predicting patient survival at one year **(A)** and 3 years **(B)** in extension cohort.

A nomogram was developed to predict one-year and three-year overall survival (OS) for TNBC patients based on factors from the training cohort (**Figure 5**). The model’s predictive accuracy was assessed using the C-index, AUC, and calibration curves. The C-index was 0.853 (95% CI: 0.749-0.957) in the training cohort and 0.854 (95% CI: 0.750-0.958) in the validation cohort. AUC values for one-year and three-year OS were 0.908 and 0.834 for the training cohort, and 0.906 and 0.836 for the validation cohort, respectively (**Figure 6**). Calibration curves confirmed strong alignment between predicted and actual outcomes, validated by the Bootstrap method (**Figure 7**). The model was further tested in an extension cohort of 163 late-stage TNBC patients, yielding a C-index of 0.703 (95% CI: 0.599–0.807) and consistent calibration (**Figure 8**).

**Figure 9:**
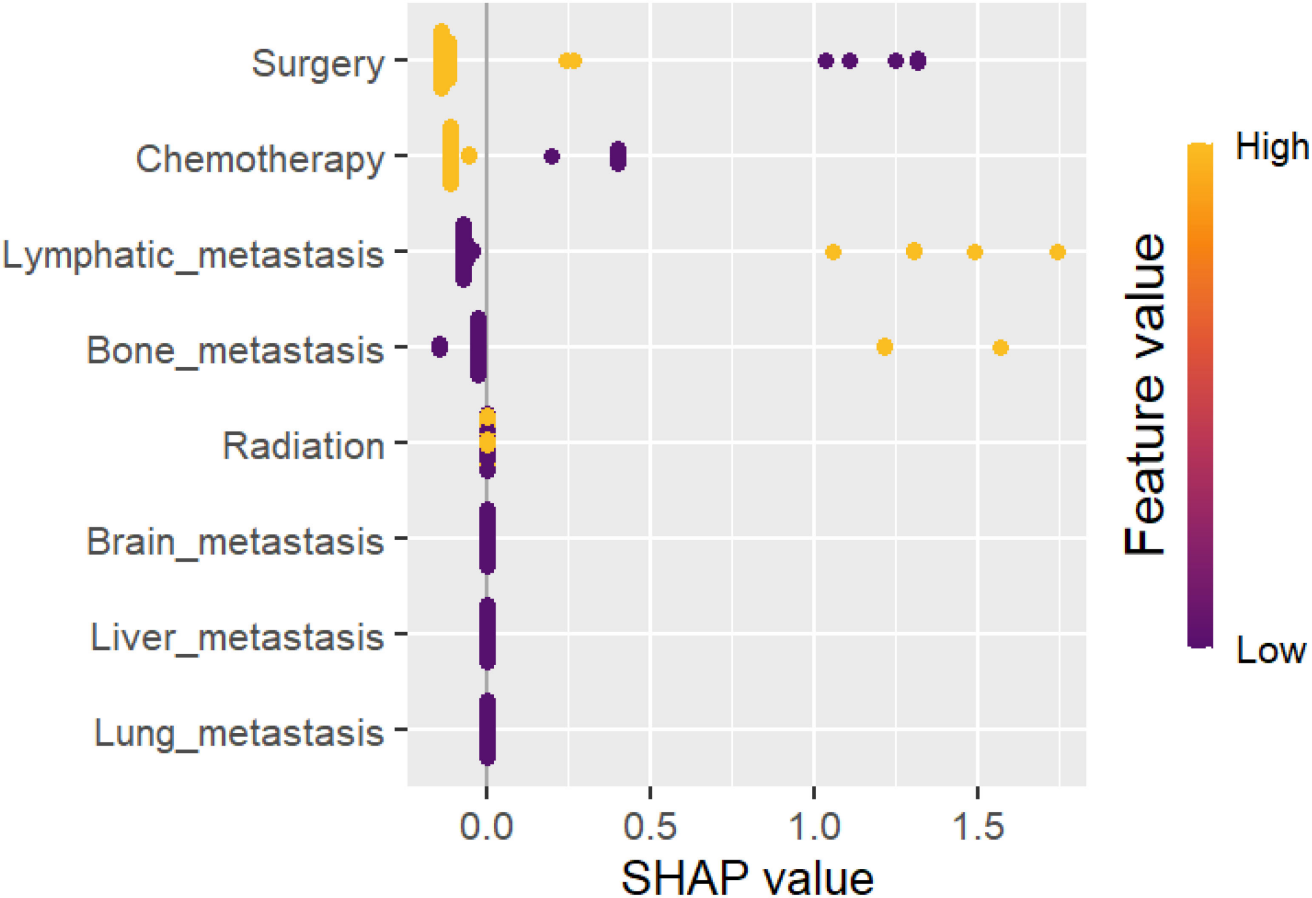
Summary plots for SHAP values. The overall survival rate is presented herewith. For each predictor, a single point represents a single patient, with the x-axis denoting the impact of the feature on the model’s output for that particular patient. A positive SHAP value is indicative of an increased risk of mortality, whereas a negative value is associated with an improved prognosis. The predictors are ordered along the y-axis in accordance with their ranking. The position of a feature on the plot indicates its relative significance in the model, with the higher-ranking features positioned higher on the plot. For the TNBC, Our work outcomes have major clinical and scientific ramifications. The importance of early tumor excision, specifically in localized stages of the disease, is shown by the substantial correlation (HR: 0.298) among surgical resection and better survival rates. The survival advantages of radiation (HR: 0.607) and chemotherapy (HR: 0.442) highlight their critical functions in controlling metastatic disease and in perioperative care. The main need of early detection and efficient systemic therapy is further addressed by the discovery that distant metastases (HR: 1.743) are a strong predictor of unfavorable outcomes. Clinicians can utilize our outcomes to customize treatment methods as per the stage of the disease and metastases. The results also support policy metrics that improve the access to multimodal treatments and support early screening. From a scientific view, integrating Explainable AI with ML models lays the major base work for generating predictive models that are more individualized. This strategy opens the door for further research to examine cutting-edge treatment alternatives and ensure the occurred outcomes in a range of demographics.It is significant to identify the several difficulties and limitations of our work has. The completeness of the data is a primary concern because the outcomes may have been affected by missing data on histological grading and particular clinical factors. Additionally, the experimental outcomes may not be as widely applicable to other populations due to regional disparities among the SEER and Fujian Cancer Hospital cohorts, such as differences in patient demographics, healthcare practices, and treatment accessibility. Furthermore, possible confounders that may affect prognostic results were not taken into observation, such as socioeconomic characteristics, genetic alterations (e.g., BRCA status), and variations in chemotherapy regimens. Notwithstanding these issues, our study outcomes are more robust and applicable due to the high sample size, the use of ML techniques, and the inclusion of an extension cohort. By applying more precise datasets and examining other variables that can affect the prognosis of triple-negative breast cancer (TNBC), future studies should address the existing limitations.

A summary of the SHAP values for the ten most significant predictors of overall survival (OS) is presented in **Figure 6**. The most significant predictor of overall survival was surgical resection of the primary tumor, followed by chemotherapy, lymphatic metastasis, bone metastasis and radiation therapy. (See **Figure 9**).

## 4 Discussion

The characteristics of triple-negative breast cancer (TNBC), including high heterogeneity, aggressiveness, and poor prognosis, present significant challenges in treatment strategies (2, 15). Despite advancements in novel therapies, the value of traditional treatments such as surgery, chemotherapy, and radiotherapy remain critical. This study utilized the SEER database to construct a column-line chart predicting overall survival (OS) in TNBC patients. The analysis confirmed key prognostic factors and emphasized the continued importance of these interventions in the era of personalized oncology.

Cox regression analysis identified advanced age, bilateral breast cancer, distant metastases, and later TNM stages as independent risk factors for poor survival outcomes (16, 17). Conversely, treatments such as surgery, radiotherapy, and chemotherapy significantly improved OS, as corroborated by previous research (4, 18). A multivariate analysis highlighted the critical role of surgical resection, with a hazard ratio of 0.298, indicating its protective effect against mortality (8).

Column-line plots developed in this study demonstrated robust predictive accuracy for 1- and 3-year survival probabilities, with a C-index of 0.854 in the validation cohort. These findings align with other studies showcasing the utility of nomograms in oncology (12, 14). Calibration curves further confirmed the reliability of predictions, strengthening the clinical applicability of this approach.

The integration of explainable AI (XAI) and SHAP values allowed for transparent identification of influential prognostic factors, such as tumor size, lymphatic involvement, and distant metastases (19, 20). This interpretability is essential for clinicians aiming to stratify patients based on risk and tailor treatment plans effectively.

Although this study contributes valuable insights, it is not without limitations. The retrospective design may introduce selection bias, and the lack of genomic data prevents a deeper understanding of TNBC molecular subtypes (Marr et al., 2020; 21). Additionally, external validation in non-American populations is necessary to generalize findings (22).

Future research should incorporate genomic and transcriptomic data to refine prognostic models further. Expanding the study cohort to include perioperative patients and evaluating novel therapeutic approaches, such as immune checkpoint inhibitors, can enhance the clinical relevance of predictive analytics in TNBC (11, 23). By addressing these areas, predictive models can evolve to provide even more precise and personalized care for TNBC patients.

From a policy standpoint, these results underscore the potential for developing AI-driven clinical decision support tools tailored for TNBC management. Such tools can aid healthcare systems in optimizing resource allocation and improving patient care outcomes. By utilizing this model, healthcare institutions can refine existing treatment guidelines, prioritize high-risk patients more effectively, and enhance the overall efficiency and efficacy of oncology care delivery.

The protective impact of surgical resection (HR: 0.298) highlights its major role in enhancing survival, specifically in localized TNBC. However, combining this result with novel therapeutic methods is critical for optimizing treatment results. Latest advancements like immune checkpoint inhibitors and targeted therapies such as PARP inhibitors, are reshaping TNBC management, particularly for advanced and metastatic cases. Integrating surgery with immunotherapy may improve immune responses by lessen the tumor burden, while perioperative chemotherapy can further enhance long-term survival. Moreover, the emergence of antibody-drug conjugates (ADCs) gives more options for patients with distinctive type of metastases. Additionally, our work should explore the synergy among surgical resection and these progressive treatments, focusing to enhance both local control and systemic outcomes, ultimately advancing personalized care for TNBC patients.

The predictive accuracy of the column-line plots (C-index: 0.854) shows their capability for real-world clinical utilities. By combining key prognostic metrics like tumor size, lymph node involvement, and distinctive metastases, these plots provides clinicians an intuitive tool for predicting one year and three year survival probabilities. Their visual and quantitative nature provides shared decision-making, enhancing personalized treatment planning based on individual patient’s risk profiles. For instance, patients identified as high-risk would be prioritized for advanced and aggressive treatments, while low-risk patients would benefit from less intensive approaches, minimizing treatment-related side effects. Additionally, the simple nature of column-line plots helps their combination clinical workflows, bridging the gap among complex ML models and real-time oncology applications.

The predictive nomograms are implemented in our work can considerably impact clinical approaches by assisting routine patient examination and personalized treatment scheduling. By integrating key prognostic metrics like tumor size, lymph node involvement, metastases, and patients’ treatment history, these nomograms offer a quick and practical method for estimating one year and three-year survival probabilities. Their intuitive development permits medical experts to evaluate individual patient risk, instructing decisions on surgery, chemotherapy, and radiotherapy. For instance, high-risk patient info gathered by the nomogram might advantage from advanced and aggressive treatment regimens, while patients with low risk may stop overtreatment, lessen the side effects. Combining the proposed tools into clinical workflows can improve early intervention, enhance treatment outcomes, and assist evidence-based decision-making, finally improvising personalized care in TNBC management.

## 5 Conclusion

In conclusion, the development of novel therapies and refined typing approaches for triple-negative breast cancer (TNBC) has improved its prognosis, though it still remains one of the more challenging subtypes of breast cancer. This study analyzed the SEER database to identify prognostic factors, construct a predictive survival model, and evaluate its performance, validation, and feature importance. Surgical intervention was identified as the most significant predictor of survival, followed by chemotherapy, lymphatic and bone metastasis, and radiotherapy. These findings support personalized treatment strategies, optimizing clinical decisions, and patient stratification for future trials. This model leverages explainable AI and machine learning to predict survival with high accuracy, offering real-world applications for patient risk assessment and tailored treatment plans. By incorporating factors like marital status and emerging treatments (e.g., immunotherapy, angiogenesis inhibitors), future models can further refine survival predictions. The work also suggests that separate models for perioperative and late-stage patients can enhance therapeutic precision.

The predictive model developed serves as a valuable resource for clinicians by providing a structured approach to stratifying patients with TNBC. This enables healthcare providers to make more informed and tailored treatment decisions based on individual risk profiles. For example, patients exhibiting high-risk characteristics—such as extensive involvement of lymph nodes, evidence of distant metastases, or aggressive tumor histopathology can be prioritized for more intensive treatment regimens that would include aggressive chemotherapy and immunotherapy. Conversely, patients categorized as low-risk may be spared from unnecessary aggressive treatments, thus minimizing exposure to potential toxic side effects. Moreover, this model aids in the efficient allocation of healthcare resources by identifying patients who require more intensive follow-up and systemic therapy, ensuring that clinical resources are utilized optimally and effectively.

However, limitations include incomplete data on chemotherapy regimens, genetic factors (e.g., BRCA mutations), and marital status. The external validity of the model requires further validation in diverse populations, and the model’s applicability to perioperative patients needs further refinement. Despite these limitations, the study provides a promising step toward improving TNBC treatment through personalized, data-driven approaches. Adding genomic data into future prognostic approaches may majorly enhance TNBC stratification and personalized care. Genomic metrics like BRCA1/2 mutations, TP53 alterations, and immune gene expressions have shown solid associations with TNBC prognosis and treatment responses. By adding these biomarkers, future ML models can improve risk prediction and support tailored therapeutic approaches like PARP inhibitors for BRCA-mutated tumors or immune checkpoint inhibitors for tumors with high PD-L1 expression. Moreover, multi-omics data combination, integrating genomic, transcriptomic, and clinical variables, might additionally filter patient stratification, providing advanced precise predictions and optimizing treatment choices for both early-stage and metastatic TNBC.

## Data Availability

The original contributions presented in the study are included in the article/supplementary material. Further inquiries can be directed to the corresponding authors.
