# Correction: Analysis of prognostic factors and nomogram construction for postoperative survival of triple-negative breast cancer

**DOI:** 10.3389/fimmu.2025.1671587

**Published:** 2025-08-06

**Authors:** Chenxi Wang, Xiangqian Zhao, Dawei Wang, Jinyun Wu, Jizhen Lin, Weiwei Huang, Yangkun Shen, Qi Chen

**Affiliations:** ^1^ Fujian Key Laboratory of Innate Immune Biology, Biomedical Research Center of South China, College of Life Science, Fujian Normal University, Fuzhou, Fujian, China; ^2^ The Cancer Center, Fujian Medical University Union Hospital, Fuzhou, Fujian, China; ^3^ Department of Medical Oncology, Clinical Oncology School of Fujian Medical University, Fujian Cancer Hospital, Fujian Provincial Key Laboratory of Translational Cancer Medicine, Fuzhou, Fujian, China

**Keywords:** triple-negative breast cancer, SEER database, prognostic modelling, columnar plots, machine learning

In the published article, there was an error in the Funding statement: “The funder Natural Science Foundation of Fujian Province (Grant Number: 2023J011275) and Joint Funds for the innovation of science and Technology, Fujian province (Grant number: 2023Y9431)” was erroneously omitted.

The original version of this article has been updated.

